# A Rare Case of Left Anterior Descending Coronary Artery to Pulmonary Trunk Fistula Associated with Takotsubo Cardiomyopathy

**DOI:** 10.3390/diagnostics13172751

**Published:** 2023-08-24

**Authors:** Ramona Mihaela Popa, Alexandru Florin Ispas, Rosana Mihaela Manea

**Affiliations:** 1 Department of Radiology and Medical Imaging, Clinical Emergency County Hospital of Brașov, 500326 Brașov, Romania; 2 Department of Interventional Cardiology, Clinical Emergency County Hospital of Brașov, 500326 Brașov, Romania; alexandru.florin.ispas@gmail.com; 3 Faculty of Medicine, “Transilvania” University of Brașov, 500019 Brașov, Romania

**Keywords:** coronary artery fistula, invasive coronary angiography, coronary CT angiography, Takotsubo Cardiomyopathy, coronary steal, left anterior descending coronary artery, pulmonary artery trunk

## Abstract

Coronary-artery-to-pulmonary-artery fistulae represent rare vascular anomalies defined as abnormal communications between the coronary arteries and the pulmonary arterial system. Takotsubo Syndrome represents a stress-induced cardiomyopathy defined by transient regional systolic dysfunction of the left ventricle, with minimal elevation of cardiac biomarkers, without angiographic evidence of obstructive coronary artery disease. We hereby richly illustrate an unusual and rare case of a female patient with Takotsubo Cardiomyopathy and left-anterior-descending-coronary-artery-to-pulmonary-trunk fistula through multi-modality imaging evaluations, obtaining a detailed anatomical representation of the coronary arteries and the fistulous connection, which further guided the optimal treatment strategy. The patient was treated conservatively. The main teaching points of this case are the following: (1) The coronary fistula may represent just an incidental finding in a Takotsubo Cardiomyopathy clinical scenario. (2) The particularly rare association between left-anterior-descending-coronary-artery-to-pulmonary-trunk fistula and Takotsubo Cardiomyopathy presentation is mainly due to the stress-induced overstimulation of myocardial beta-1 receptors, accentuating the coronary steal phenomenon in the setting of the coronary fistula, manifesting as anginal pain, and also the stress-induced adrenergic drive causing the Takotsubo-like presentation with apical ballooning of the left ventricle.

**Figure 1:**
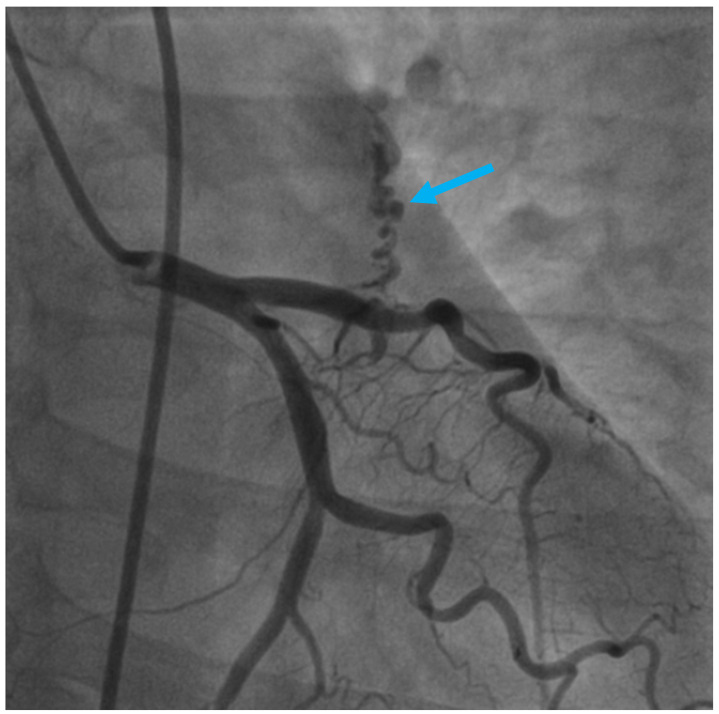
Invasive coronary angiography of a 54-year-old female patient who was admitted to the emergency department with typical angina related to high emotional stress, moderately relieved after sublingual nitrates.

Regarding her medical history, the patient was not known to have any cardiovascular diseases. No alcohol or drug use was noted. The only cardiovascular risk factor included was smoking (for 15 years). The patient was not under any pharmacological treatment at the time of admission.

On cardiovascular examination, the following were noted: slight displacement of the apical impulse of the left ventricle, tachycardia, no jugular vein distention. Pulses were intact bilaterally in upper and lower extremities. Lungs were clear of auscultation bilaterally. Oxygen saturation = 95% on room air. Blood pressure = 161/82 mmHg. Respiratory rate = 25 breaths/minute. Temperature = 36.5 °C.

Electrocardiogram showed normal sinus rhythm, heart rate = 94 beats/min, QRS ax +60°, and ST segment elevations V4–V6 (2 mm).

Laboratory investigations showed slightly elevated levels of CK-MB and troponin–I (due to the myocardial stunning as a result of catecholaminergic effect). Full blood count, lipid profile, electrolytes, and renal and hepatic function were normal.

Transthoracic echocardiography (Video S1) showed systolic “ballooning” of the left ventricular apex and akinesis of the apex and hypokinesis of the mid segments of all left ventricular walls—highly suggestive of a Takotsubo Cardiomyopathy presentation.

Other ultrasonographic findings include:

- LVEF < 50%;
- Grade II aortic regurgitation;
- Grade II mitral regurgitation;
- Right ventricle with normal longitudinal function;
- No signs of secondary pulmonary hypertension;
- No pericardial effusion.

The patient was referred to the cardiac catheterization laboratory to undergo invasive coronary angiography.

Invasive coronary angiography revealed a small-caliber anomalous tortuous vascular communication (marked with the blue arrow) arising from the proximal segment of the left anterior descending coronary artery (LAD), highly suggestive of a coronary fistula (Figure 1; Videos S2 and S3).

Additionally, the left main artery (LMA) and the left circumflex artery (LCX) present normal caliber; no significant stenosis was noted.

Furthermore, coronary catheterization also showed normal caliber of the right coronary artery (RCA), the posterior descending artery (PDA), the postero-lateral branch (PLB), and the acute marginal artery. No significant coronary stenosis was noted (Video S4). Cardiac computed tomography (CT) was performed and revealed the following imaging findings, richly illustrated in Figure 2, Figure 3 and Figure 4. 

Furthermore, presenting a very tortuous and diffuse vascular path which could not be resolved with interventional procedures (percutaneous transcatheter closure), our patient was managed conservatively, and symptoms completely resolved after optimal medical treatment, not needing further surgical ligature. Cardiac biomarkers, LVEF, and ECG changes normalized. No complications during hospitalization were noted.

Patient remained asymptomatic at 1-month and 6-months follow-ups.

Coronary artery fistulae represent congenital or acquired vascular connections between the coronary arteries and the cardiac chambers or other vascular structures (such as superior vena cava, pulmonary arteries/veins), bypassing the myocardial capillary network [1,2,3,4].

Most of these fistulous communications are congenital, arising from the right coronary artery (50–60%) and the left anterior descending artery (25–42%), followed by the circumflex coronary, diagonal and left main coronary artery (18.3%, 1.9%, and 0.7%, respectively) [5]. Coronary artery fistulae can also be acquired from trauma, congenital heart surgery, transcutaneous techniques (myocardial biopsy), percutaneous coronary intervention, coronary stent placement, coronary bypass surgery, chest irradiation or Kawaski disease [5,6,7]. Coronary vasculitis and myocardial infarction may also lead to coronary artery fistulae in the chronic phases [8].

The embryologic explanation still remains the Hackensellner involution–persistence hypothesis, which refers to the following aspects: in the normal scenario, only two of six branches from the aortic sinus remain to form the coronary arteries, and there is involution of the rest of the branches [3,9,10]. In case the involution of the branches fails, a coronary-artery-to-pulmonary-artery fistula is formed, meaning the branch from the pulmonary sinus, which is otherwise involuted, abnormally persists and connects to the normal branches from the aortic sinus coronary arteries [3,9,10].

The majority of coronary artery fistulae (89%) drain into the pulmonary trunk rather than into other segmental pulmonary arteries [3].

There are two types of coronary-artery-to-pulmonary artery fistulae:

1. The first type is represented by a single fistulous communication between the left anterior descending artery or the right coronary artery and the main pulmonary trunk [2,3].
2. The other type involves multiple small-caliber fistulous connections from the left anterior descending artery or right coronary artery, which drain into the main pulmonary trunk [2,3]. It can also terminate as a maze pattern of fine vessels, forming a plexus with extensive mural distribution [5].

It is of the utmost importance to note that coronary artery fistula with a single fistulous connection is more likely to involve hemodynamic disturbance-related symptoms rather than a fistula with multiple vascular connections [3].

According to the angiographic classification of Sakakibara et al., there are two main categories of coronary artery fistulae:

- Type A—proximal coronary dilation at the origin of fistula and normal distal end;
- Type B—coronary dilatation over the entire length [5,11].

Coronary artery fistulas present a broad spectrum of clinical presentations, ranging from mild non-specific symptoms to affecting the hemodynamic parameters and leading to complications, including myocardial ischemia, heart failure, arrhythmia or even sudden cardiac death [3,11,12,13]. Despite the varieties in clinical features of this rare anomaly, a Takotsubo Cardiomyopathy scenario is rarely a presenting feature or an associated condition. A coronary steal syndrome represents a possible mechanism induced by the fistula, leading to regional wall ischemia and resulting in a TM-like picture [5,14,15,16,17].

A coronary steal phenomenon refers to the diversion of blood away from normal myocardial circulation, further leading to (reversible) myocardial ischemia, mimicking a Takotsubo-Cardiomyopathy-like presentation [5,14,15,16,17]. Additionally, coronary vasospasm and abnormalities in coronary microvascular function and catecholamine-mediated cardiotoxicity, triggered by intense emotional-stress-induced adrenergic drive playing a dominant role in the pathophysiological mechanism [5,14,15,16,17]. High levels of circulating epinephrine stimulate beta-2-adrenergic-receptors in ventricular myocytes, causing a switch from Gs protein to Gi protein, which further blocks the pro-apoptotic effect of intense beta-1-stimulation. Moreover, Gi protein is negatively inotropic, an effect which is dominant at the cardiac apex due to maximum beta adrenergic receptor density [5,14,15,16,17].

Nevertheless, more case reports and studies regarding this particular topic are needed in the current literature and should be encouraged in order to improve further knowledge and deeper understanding of this possible interrelation between these two pathological entities.

Despite the clinical varieties regarding coronary fistulae (depending on the fistula’s type, caliber, vascular connection, etc.) Takotsubo Cardiomyopathy is rarely included as a presenting feature or as an associated condition. A possible mechanism is considered the steal phenomenon induced by the coronary fistula, leading to regional wall ischemia and resulting in a TM-like picture [5,14,15,16,17].

However, further research in this scientific direction could lead to thorough knowledge of the pathophysiological mechanism and could ultimately contribute to an optimal treatment strategy in each particular case, further reducing the rates of morbidity and mortality.

## Figures and Tables

**Figure 2:**
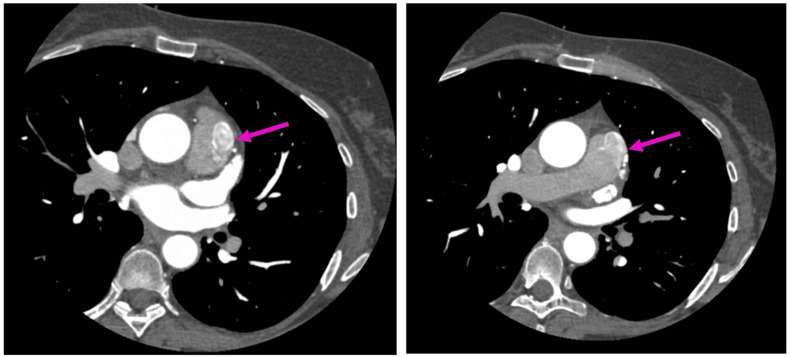
Axial sections of coronary CTA acquisitions—entitled “A picture is worth a thousand words”. show an anomalous tortuous vascular connection with drainage situs in the anterior surface of the pulmonary trunk, fully illustrating diffusion of the resulting contrast blush within the pulmonary trunk, also known as the contrast shunt sign, in a relatively less-opacified pulmonary trunk (pink arrow).

**Figure 3:**
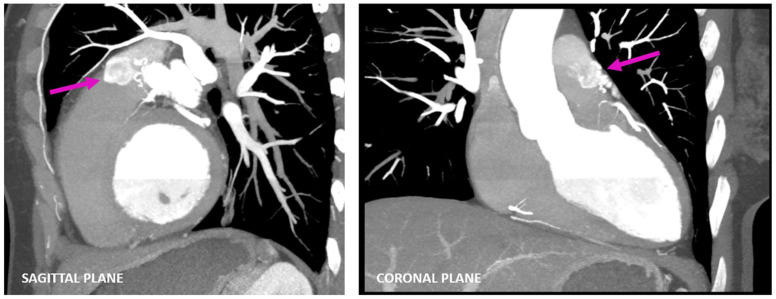
Multiplanar images (sagittal and coronal planes) of coronary CTA acquisitions also visualized using maximum-intensity projection representing a faint flow of contrast from the fistulous connection into the pulmonary trunk—the contrast shunt sign (pink arrow).

**Figure 4:**
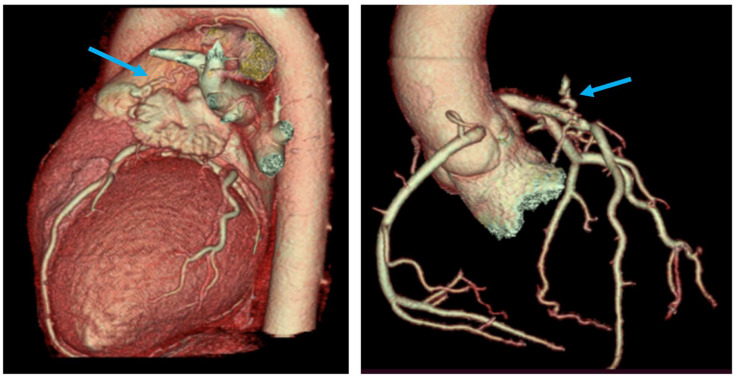
Coronary CT angiography (CTA)—three-dimensional volume-rendering images vividly depict the presence of a fistulous connection between the proximal segment of the LAD and the pulmonary trunk (marked with the blue arrow). Coronary arteries present anatomic origins of the right and left sinuses of Valsalva and coronary co-dominance. No significant coronary artery stenoses were visualized.

## Supplementary Materials

The following supporting information can be downloaded at: https://www.mdpi.com/article/10.3390/diagnostics13172751/s1.
